# Racial disparities in COVID-19 outcomes exist despite comparable Elixhauser comorbidity indices between Blacks, Hispanics, Native Americans, and Whites

**DOI:** 10.1038/s41598-021-88308-2

**Published:** 2021-04-22

**Authors:** Fares Qeadan, Elizabeth VanSant-Webb, Benjamin Tingey, Tiana N. Rogers, Ellen Brooks, Nana A. Mensah, Karen M. Winkfield, Ali I. Saeed, Kevin English, Charles R. Rogers

**Affiliations:** 1 Department of Family and Preventive Medicine, University of Utah School of Medicine, 375 Chipeta Way, Suite A, Salt Lake City, UT 84108 USA; 2 Sorenson Impact Center, University of Utah—David Eccles School of Business, Salt Lake City, UT USA; 3 Meharry-Vanderbilt Alliance, Department of Radiation Oncology, Vanderbilt University Medical Center, Nashville, USA; 4 Department of Internal Medicine, Meharry Medical College, Nashville, TN USA; 5 Norton Thoracic Institute, St. Joseph’s Hospital and Medical Center, Phoenix, AZ USA; 6 Albuquerque Area Southwest Tribal Epidemiology Center, Albuquerque, NM USA

**Keywords:** Diseases, Risk factors

## Abstract

Factors contributing to racial inequities in outcomes from coronavirus disease 2019 (COVID-19) remain poorly understood. We compared by race the risk of 4 COVID-19 health outcomes––maximum length of hospital stay (LOS), invasive ventilation, hospitalization exceeding 24 h, and death––stratified by Elixhauser comorbidity index (ECI) ranking. Outcomes and ECI scores were constructed from retrospective data obtained from the Cerner COVID-19 De-Identified Data cohort. We hypothesized that racial disparities in COVID-19 outcomes would exist despite comparable ECI scores among non-Hispanic (NH) Blacks, Hispanics, American Indians/Alaska Natives (AI/ANs), and NH Whites. Compared with NH Whites, NH Blacks had longer hospital LOS, higher rates of ventilator dependence, and a higher mortality rate; AI/ANs, higher odds of hospitalization for ECI = 0 but lower for ECI ≥ 5, longer LOS for ECI = 0, a higher risk of death across all ECI categories except ECI ≥ 5, and higher odds of ventilator dependence; Hispanics, a lower risk of death across all ECI categories except ECI = 0, lower odds of hospitalization, shorter LOS for ECI ≥ 5, and higher odds of ventilator dependence for ECI = 0 but lower for ECI = 1–4. Our findings contest arguments that higher comorbidity levels explain elevated COVID-19 death rates among NH Blacks and AI/ANs compared with Hispanics and NH Whites.

## Introduction

Severe acute respiratory syndrome coronavirus 2 (SARS-CoV-2), the virus that causes COVID-19, has disproportionately affected counties across the United States (US) that have substantially more racially and ethnically diverse populations^1,2^. Total deaths from COVID-19 in the US have eclipsed 540,000 (as of March 24, 2021)^3^, with the highest mortality occurring among non-Hispanic (NH) Blacks and American Indians/Alaska Natives (AI/ANs), whose mortality rates are 1.9 and 2.4 times higher, respectively, than those of NH Whites (as of March 12, 2021)^4^.

A confluence of social, economic, and biologic factors, together with a higher prevalence of comorbidities in AI/AN, Hispanic/Latino, and NH Black communities, has resulted in a greater COVID-19 burden and worse outcomes among medically underserved and minority populations^2^. According to the Centers for Disease Control and Prevention (CDC), comorbidities such as cardiovascular diseases, cancer, and obesity present some of the strongest and most consistent evidence for risk of hospitalization, intensive care unit admission, need for ventilation, and death due to COVID-19^5^. The higher prevalence of comorbidities experienced by Hispanics/Latinos, NH Blacks, and AI/ANs may account for why these populations are, respectively, 3.1, 2.9, and 3.7 times more likely than NH Whites to be hospitalized for COVID-19^4^. NH Blacks are more likely to require mechanical ventilation^6^. Despite similar median lengths of hospital stay across racial/ethnic groups^7,8^, and despite race not being associated with an increased risk of in-hospital death from COVID-19^9^, minority populations often experience twice the mortality rate of NH Whites^6,10^. While studies of other respiratory infectious diseases such as influenza, specifically H1N1 influenza, have suggested links between race and worse outcomes^11,12^, the widespread nature of the COVID-19 pandemic also suggests that factors independent of underlying health conditions may be contributing to COVID-19 severity in the US.

The increased burden of comorbidity among NH Blacks^13,14^ is hypothesized to be a major contributing factor to adverse COVID-19 outcomes^15,16^, including an increased risk of death^17,18^. However, both single-site and multisite studies report that disparities in COVID-19 hospitalizations and deaths among NH Blacks persist after adjustment for comorbid conditions^7,19,20^. We hypothesize that racial disparities in COVID-19 outcomes exist despite comparable Elixhauser comorbidity index (ECI) scores among AI/ANs, NH Blacks, Hispanics/Latinos, and NH Whites.

We used the ECI^21^ to further interrogate COVID-19 disparities and objectively ascertain the burden of comorbid conditions on COVID-19 health outcomes. The ECI encompasses 31 diagnoses, including cardiovascular disease, diabetes, liver disease, and pulmonary disease, each weighted by mortality risk. A total ECI score is generated from the sum of individual weights; a higher score indicates a higher burden of comorbidity^21,22^. Studies with sample sizes ranging from 574 to more than 14,000,000 have established the ECI’s validity as a prognostic indicator^23,24^.

Prior studies using the Charlson^25^ and Elixhauser comorbidity indices to account for comorbid conditions in the context of COVID-19 have (1) failed to account for racial disparities^26^, (2) used data from single sites or single hospital systems^19,27–29^ or (3) failed to capture other relevant COVID-19 health outcomes beyond death and hospitalization (e.g., length of hospital stay^30^ [LOS], need for ventilation^31,32^). Our study therefore aimed to evaluate 4 COVID-19 health outcomes stratified by ECI ranking: hospitalizations exceeding 24 h, maximum LOS, ventilation, and death.

## Methods

### Settings

We used data from the Cerner COVID-19 De-Identified Data cohort, a subset of the Cerner Real-World Data cohort. Data in Cerner Real-World Data is extracted from the electronic health records (EHRs) of hospitals with which Cerner has a data use agreement and may include pharmacy, clinical and microbiology laboratory, and admission data, as well as billing information from affiliated patient-care locations. All admissions, medication and dispensing orders, laboratory orders and specimens are date and time stamped, providing a temporal relationship between treatment patterns and clinical information. Cerner Corporation has established Health Insurance Portability and Accountability Act (HIPAA)–compliant operating policies to establish de-identification for Cerner Real-World Data^33,34^. EHR data are cleaned, standardized, and person-matched before being completely de-identified per HIPAA standards. Records of patients identified as having an encounter associated with a diagnosis of or a recent (up to 2 weeks prior) positive lab test for COVID-19 between January and June 2020 were included in the COVID-19 data set. To assess possible disease histories, all encounters and additional medical information for this patient cohort are collected, extending as far back as January 1, 2015, where available. A total of 62 health systems across the US contributed records to this data set.

The University of Utah Institutional Review Board (IRB #136696) determined that this study did not meet the definition of human subjects research according to federal regulations because (1) the investigators used secondary data and did not collect data through intervention or interaction with an individual, and (2) no personally identifiable information was captured in the data. The IRB also determined that the study did not meet the US Food and Drug Administration’s (FDA’s) definition of human subjects research because it did not involve a drug, device, or any other FDA-regulated product. Thus, the IRB waived the requirements for ethical approval and informed consent for this study.

### Measurements

The outcomes of interest involved 4 indications of clinical complications in patients with COVID-19: hospitalization, maximum hospital LOS, invasive ventilator dependence, and death. These indications were constructed from EHR data to reflect a unique risk profile per patient. Additionally, every outcome had to involve a COVID-19 diagnosis or laboratory indication.

We measured maximum LOS by calculating the difference in days between the start and end dates of each patient encounter and taking the maximum difference per patient. Hospitalization was a binary indicator of whether a patient ever had an LOS of 1 day or more. Invasive ventilator dependence was a binary indicator of whether a patient ever had a diagnosis, procedure, encounter, result, or indication signifying reliance on an invasive ventilator. The full list of code types (Current Procedural Terminology [CPT], International Classification of Diseases [ICD], Logical Observation Identifiers Names and Codes [LOINC], and Systematized Nomenclature of Medicine—Clinical Terms [SNOMED CT]) and the corresponding codes used to define invasive ventilator dependence are found in Supplemental Table 1. These codes were kept separate from indications of less-severe ventilator dependence. Death was a binary indicator of whether a patient died at discharge or any time thereafter until the time of data collection. For additional analyses, in-hospital death was obtained and restricted to death at discharge (excluding any later deaths occurring outside of the hospital).

The predictors of interest were race (AI/AN, Asian/Pacific Islander [API], NH Black/African American, White, other/unknown race); ethnicity (Hispanic or Latino); and a comorbidity score derived from the ECI. Like the Charlson comorbidity index (CCI)^18^, the ECI measures patient comorbidity by calculating a risk-assessment score based on ICD-10 diagnosis codes. However, the ECI considers more chronic disease indications (with some more relevant to COVID-19 complications) than does the CCI (31 vs. 17)^35^ The ECI is weighted using the Agency for Health Care Research and Quality (AHRQ) methodology^36^ and scores are grouped into categories of less than 0, 0, 1–4, and 5 or higher^24^. A full list of the diseases involved in the score calculation and the corresponding ICD-10 codes is found in Supplemental Table 2^37^. Other demographic characteristics included for analysis were sex, insurance status, and 1-digit zip-code region (categorical variables) and age in years (a continuous variable).

### Statistical analysis

Overall demographic characteristics were presented for patients in the COVID-19 cohort. Categorical variables were expressed by frequencies and percentages. Because continuous variables were not normally distributed, they were expressed as medians and interquartile ranges (IQRs). These characteristics were also stratified by ECI group to assess significant demographic differences across comorbidity groups. Categorical variables were compared using a chi-square test and nonparametric continuous variables by a Kruskal–Wallis rank sum test. Each outcome was presented across the demographic and clinical characteristics of interest: gender, race/ethnicity, insurance status, and ECI group. Medians (IQRs) were presented for maximum LOS and frequencies (percentages) for hospitalization, invasive ventilator dependence, and death.

To determine the adjusted associations of race/ethnicity and comorbidity with outcomes, multi-level regression models were fit using logistic regression models for hospitalization, invasive ventilator dependence, and death. Because LOS followed a continuous, exponential distribution, an exponential regression model was fit for maximum LOS. Adjusted odds ratios with 95% confidence intervals (CIs) were reported for the logistic model predictors. Adjusted exponentiated coefficients relating to the percentage change in expected maximum LOS with 95% CIs were reported for the exponential model predictors. All models were fit with race/ethnicity and ECI score and adjusted for age, sex, and insurance status. Additionally, models involved a random effect of 1-digit zip-code to account for clustering of results in similar regions. The predictive ability of the models was assessed for both logistic and exponential models. For logistic regression models, an area under the receiver operating characteristic curve (AUC) was calculated to assess the models’ ability to correctly classify outcome categories. For the exponential model, the coefficient of determination (R^2^) was calculated to estimate the percentage of variation in LOS as explained by the model predictors.

To assess the adjusted impact of race/ethnicity and comorbidity on the hazard of death, a Cox proportional hazards regression model was fit and adjusted for all variables included in the previous models. The outcome involved both time (from hospital admission to hospital discharge) and indication of in-hospital death (dead or alive at discharge).Adjusted hazard ratios (aHRs) and 95% CIs were reported. For all models, diagnostics were performed to ensure optimal model fit.

To further assess differences across comorbidities, sub-analyses were performed by stratifying the cohort by ECI groups (less than 0, 0, 1–4, 5 or higher) and running the same models within each group. Additionally, scatterplot figures were constructed to show the impact of race/ethnicity and comorbidity on the predicted outcomes of clinical complications. Each figure showed the predicted outcome against the ECI score. Smoothed lines were fit amongst the data by generalized additive regression models with shrinkage cubic-regression splines. This was done by fitting different lines for the different racial/ethnic groups. All hypothesis tests were 2-sided with a significance level of 5%. R version 3.6.1 (R Foundation for Statistical Computing, Vienna, Austria) was used for all analyses. In addition, R package “comorbidity” (version 0.5.3) was used to calculate comorbidity scores.

### Sample size calculation

**Figure 1:**
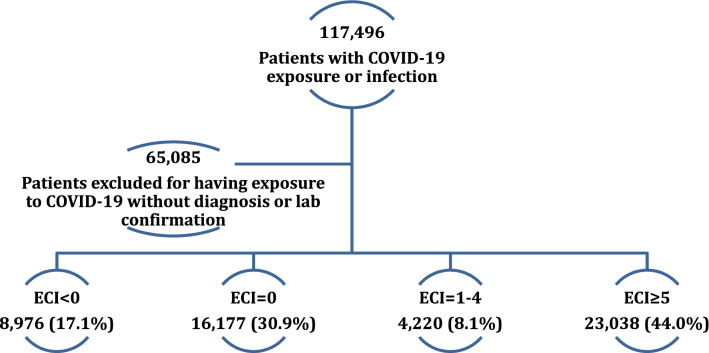
Data flow chart for the study. The final cohort size of 52,411 COVID-19 patients is stratified by ECI group.

Using 80% power, the stratified race/ethnicity distribution by Elixhauser AHRQ-weighted comorbidity group (Table 1), and the risk of COVID-19 complications by race/ethnicity (Table 2), we needed a sample size of at least 3,591 subjects for each ECI category, assuming the most stringent comparison between AI/AN and NH Whites, to achieve a small effect size^38^ of OR = 1.68 in a 2-sided examination. This sample size was attainable in our study given that we had a total of 52,411 subjects (8976; 16,177; 4220; and 23,038 for ECI groups less than 0, 0, 1–4, and 5 or higher, respectively), as shown in the data flow chart (Fig. 1).

**Table 1:** Demographic and clinical characteristics of COVID-19 infected patients by Elixhauser AHRQ-weighted comorbidity Index and overall.

	Total n (%^a^)	Elixhauser AHRQ-weighted comorbidity group				
		< 0	0	1–4	≥ 5	*p* value^f^
		n (%^a^)	n (%^a^)	n (%^a^)	n (%^a^)	
Comparison	52,411 (100.00)	8976 (17.1)	16,177 (30.9)	4220 (8.1)	23,038 (44.0)	
**Age (Years)**^**b**^	53 (35–68)	51 (37–62)	32 (20–47)	46 (28–61)	64 (48–77)	**< 0.001**^**g**^
**Gender**						**< 0.001**
Female	26,512 (50.6)	4902 (54.6)	8206 (50.7)	2354 (55.8)	11,050 (48.0)	
Male	25,800 (49.2)	4053 (45.2)	7950 (49.1)	1857 (44.0)	11,940 (51.8)	
Other^c^	99 (0.2)	21 (0.2)	21 (0.1)	9 (0.2)	48 (0.2)	
**Race and ethnicity**						**< 0.001**
Non-Hispanic American Indian or Alaska Native	1070 (2.0)	179 (2.0)	503 (3.1)	72 (1.7)	316 (1.4)	
Non-Hispanic Asian or Pacific Islander	1447 (2.8)	208 (2.3)	401 (2.5)	98 (2.3)	740 (3.2)	
Non-Hispanic Black or African American	10,667 (20.4)	2200 (24.5)	2429 (15.0)	954 (22.6)	5084 (22.1)	
Non-Hispanic White	15,048 (28.7)	2141 (23.9)	3197 (19.8)	1156 (27.4)	8554 (37.1)	
Non-Hispanic Other^d^	5754 (11.0)	877 (9.8)	2236 (13.8)	381 (9.0)	2260 (9.8)	
Hispanic or Latino	18,425 (35.2)	3371 (37.6)	7411 (45.8)	1559 (36.9)	6084 (26.4)	
**Insurance**						**< 0.001**
Private	18,015 (34.4)	3678 (41.0)	7129 (44.1)	1687 (40.0)	5521 (24.0)	
Government/misc	1853 (3.5)	312 (3.5)	676 (4.2)	138 (3.3)	727 (3.2)	
Medicaid	8597 (16.4)	1837 (20.5)	2936 (18.1)	782 (18.5)	3042 (13.2)	
Medicare	11,791 (22.5)	1262 (14.1)	929 (5.7)	743 (17.6)	8857 (38.4)	
Self-pay	4906 (9.4)	804 (9.0)	2842 (17.6)	371 (8.8)	889 (3.9)	
Missing	7249 (13.8)	1083 (12.1)	1665 (10.3)	499 (11.8)	4002 (17.4)	
**Zip-code region**^**e**^						**< 0.001**
0	6210 (11.8)	958 (10.7)	1451 (9.0)	388 (9.2)	3413 (14.8)	
1	5593 (10.7)	1050 (11.7)	1754 (10.8)	437 (10.4)	2352 (10.2)	
2	8139 (15.5)	1468 (16.4)	1893 (11.7)	667 (15.8)	4111 (17.8)	
3	9867 (18.8)	1725 (19.2)	4552 (28.1)	978 (23.2)	2612 (11.3)	
4	2701 (5.2)	546 (6.1)	753 (4.7)	218 (5.2)	1184 (5.1)	
5	337 (0.6)	65 (0.7)	122 (0.8)	33 (0.8)	117 (0.5)	
6	1551 (3.0)	241 (2.7)	491 (3.0)	120 (2.8)	699 (3.0)	
7	3116 (5.9)	522 (5.8)	834 (5.2)	232 (5.5)	1528 (6.6)	
8	3321 (6.3)	477 (5.3)	1156 (7.1)	257 (6.1)	1431 (6.2)	
9	9012 (17.2)	1589 (17.7)	2803 (17.3)	698 (16.5)	3922 (17.0)	
Missing	2564 (4.9)	335 (3.7)	368 (2.3)	192 (4.5)	1669 (7.2)	

^a^% = column percentage.

^b^Median (Q1–Q3).

^c^Other or unknown.

^d^Other, unknown, or mixed race.

^e^0 (Connecticut, Massachusetts, Maine, New Hampshire, New Jersey, Rhode Island, Vermont), 1 (Delaware, New York, Pennsylvania), 2 (DC, Maryland, North Carolina, South Carolina, Virginia, West Virginia), 3 (Alabama, Florida, Georgia, Mississippi, Tennessee), 4(Indiana, Kentucky, Michigan, Ohio), 5 (Iowa, Minnesota, Montana, North Dakota, South Dakota, Wisconsin), 6 (Illinois, Kansas, Missouri, Nebraska), 7 (Arkansas, Louisiana, Oklahoma, Texas), 8 (Arizona, Colorado, Idaho, New Mexico, Nevada, Utah, Wyoming), 9 (Alaska, California, Hawaii, Oregon, Washington).

^f^Chi-squared test (unless otherwise noted).

^g^Kruskall–Wallis rank-sum test.

Bold indicates statistical significance at the 5% level (i.e., *p* value < 0.05).

**Table 2:** Risk of complications from COVID-19 by patient characteristics.

Comparison	Hospitalization	Maximum length of stay (days)	Invasive ventilator dependence	Deceased
	n (%^a^)	Median (IQR: Q1–Q3)	n (%^a^)	n (%^a^)
**Total**	27,774 (53.0)	1.6 (0.1–6.5)	6150 (11.7)	4695 (9.0)
**Gender**				
Female	13,307 (50.2)	1.0 (0.1–5.8)	2472 (9.3)	1962 (7.4)
Male	14,406 (55.8)	2.0 (0.1–7.2)	3664 (14.2)	2723 (10.6)
Other	61 (61.6)	2.4 (0.2–6.7)	14 (14.1)	10 (10.1)
**Race and ethnicity**				
Non-Hispanic American Indian or Alaska Native	574 (53.6)	1.9 (0.1–7.8)	236 (22.1)	113 (10.6)
Non-Hispanic Asian or Pacific Islander	876 (60.5)	2.7 (0.2–8.3)	220 (15.2)	150 (10.4)
Non-Hispanic Black or African American	6131 (57.5)	2.1 (0.2–7.5)	1383 (13.0)	1072 (10.0)
Non-Hispanic White	9811 (65.2)	3.0 (0.2–7.8)	2020 (13.4)	1998 (13.3)
Non-Hispanic other	2944 (51.2)	1.2 (0.1–6.9)	822 (14.3)	533 (9.3)
Hispanic or Latino	7438 (40.4)	0.2 (0.1–4.1)	1469 (8.0)	829 (4.5)
**Insurance**				
Private	7067 (39.2)	0.2 (0.1–3.8)	1538 (8.5)	677 (3.8)
Government/miscellaneous	957 (51.6)	1.2 (0.11–6.1)	221 (11.9)	173 (9.3)
Medicaid	4209 (49.0)	0.9 (0.1–5.1)	850 (9.9)	367 (4.3)
Medicare	9442 (80.1)	5.5 (1.9–10.9)	2213 (18.8)	2606 (22.1)
Self-pay	97 (2.0)	0.1 (0.1–0.7)	173 (3.5)	97 (2.0)
Missing	775 (10.7)	3.4 (0.2–8.8)	1155 (15.9)	775 (10.7)
**Elixhauser AHRQ-weighted comorbidity group**				
< 0	3874 (43.2)	0.3 (0.1–4.0)	440 (4.9)	195 (2.2)
0	3041 (18.8)	0.1 (0.1–0.3)	496 (3.1)	252 (1.6)
1–4	1867 (44.2)	0.3 (0.1–4.3)	313 (7.4)	190 (4.5)
≥ 5	18,992 (82.4)	5.4 (2.0–11.2)	4901 (21.3)	4058 (17.6)

^a^Row percentage.

## Results

A total of 52,411 unique patients with a COVID-19 diagnosis or recent positive laboratory result were included in the analysis cohort. The median (IQR) patient age was 53 years (35–68); 50.6% (26,512) were female. Most patients were Hispanic/Latino (18,425; 35.2%), followed by NH White (15,048; 28.7%), NH Black/African American (10,667; 20.4%), NH other or unknown race (5754; 11.0%), API (1447; 2.8%), and AI/AN (1070; 2.0%). Most had private insurance (18,015; 34.4%), followed by Medicare (11,791; 22.5%) or Medicaid (8597; 16.4%) coverage. Most lived in the southeastern US (9867; 18.8%). Forty-four percent of patients (23,038) had an ECI score of 5 or higher; 30.9% (16,177) had an ECI score of 0 (Table 1).

Table 1 also shows patient demographic characteristics stratified by ECI group. Those with higher comorbidity were older and more likely to be male, NH White, and covered by Medicare. Significant differences were observed between all demographic groups when stratified by ECI group (all *p* < 0.001).

Table 2 shows crude risk results for COVID-19-related clinical complications across patient characteristics. Compared with women, men had higher percentages of hospitalization (55.8% vs. 50.2%), a higher median LOS (2.0 vs. 1.0), higher percentages of invasive ventilator dependence (14.2% vs. 9.3%), and higher percentages of death (10.6% vs. 7.4%). NH Whites had the highest outcomes for all clinical complications except invasive ventilator dependence (hospitalization, 65.2%; median LOS, 3.0 days; death, 13.3%). AI/ANs had the highest odds of invasive ventilator dependence (22.1%). Hispanics consistently had the lowest risk of complications across all outcomes. Patients covered by Medicare and those with ECI scores of 5 or higher had the highest risk of complications across all outcomes.

Table 3 shows the association of the adjusted predictors with the 4 clinical complications of hospitalization, maximum LOS, invasive ventilator dependence, and death. (Survival modeling for time to death is presented here; logistic modeling for death is reported in Supplemental Table 3). Older patients and men (compared with women) consistently showed a higher risk of complications for all outcomes. AI/ANs had consistently higher risk of complications for all outcomes than NH Whites, all of which were significant (hospitalization aOR 1.21; maximum LOS ${e}^{\widehat{\beta }}$ 1.32; ventilator aOR 3.49; death aHR 2.06). Compared with NH Whites, APIs stayed significantly longer in the hospital (maximum LOS ${e}^{\widehat{\beta }}$ 1.15; 95% CI [1.05, 1.27]) and were significantly more likely to be ventilator dependent (aOR 1.44; 95% CI [1.22, 1.69]).

**Table 3:** Adjusted associations with hospitalization, maximum length of hospital stay, dependence on invasive ventilator, and death from COVID-19.

Variables	Hospitalization	Maximum length of stay	Invasive ventilator dependence	Deceased
	aOR^a^ (95% CI)	$e^{{\hat{\beta }}}$^b^ (95% CI)	aOR^a^ (95% CI)	aHR^c^ (95% CI)
**Age (years)**^**d**^	**1.30 (1.28, 1.32)**	**1.30 (1.29, 1.31)**	**1.16 (1.14, 1.18)**	**1.58 (1.55, 1.63)**
**Gender**				
Female	1 [Reference]	1 [Reference]	1 [Reference]	1 [Reference]
Male	**1.23 (1.18, 1.28)**	**1.22 (1.18, 1.26)**	**1.55 (1.46, 1.64)**	**1.40 (1.32, 1.49)**
Other	*1.60 (1.00, 2.57)*^f^	*1.37 (0.96, 1.95)*^f^	1.50 (0.82, 2.75)	1.35 (0.70, 2.60)
**Race and ethnicity**				
Non-Hispanic White	1 [Reference]	1 [Reference]	1 [Reference]	1 [Reference]
Non-Hispanic American Indian or Alaska Native	**1.21 (1.03, 1.43)**	**1.32 (1.16, 1.51)**	**3.49 (2.87, 4.25)**	**2.06 (1.70, 2.50)**
Non-Hispanic Asian or Pacific Islander	1.08 (0.95, 1.23)	**1.15 (1.05, 1.27)**	**1.44 (1.22, 1.69)**	1.12 (0.95, 1.33)
Non-Hispanic Black or African American	1.02 (0.95, 1.08)	**1.13 (1.08, 1.19)**	**1.31 (1.21, 1.43)**	**1.22 (1.13, 1.32)**
Non-Hispanic other	0.99 (0.91, 1.06)	1.06 (1.00, 1.12)	**1.72 (1.56, 1.90)**	**1.58 (1.43, 1.74)**
Hispanic or Latino	**0.81 (0.77, 0.86)**	**0.88 (0.85, 0.92)**	1.09 (1.00, 1.19)	**0.89 (0.82, 0.97)**
**Insurance**				
Private	1 [Reference]	1 [Reference]	1 [Reference]	1 [Reference]
Government/misc	1.09 (0.98, 1.22)	**1.11 (1.02, 1.21)**	0.93 (0.79, 1.09)	**1.51 (1.27, 1.79)**
Medicaid	**1.64 (1.54, 1.74)**	**1.65 (1.58, 1.74)**	**1.11 (1.01, 1.22)**	**1.45 (1.27, 1.65)**
Medicare	**1.51 (1.41, 1.62)**	**1.50 (1.42, 1.58)**	**0.90 (0.82, 0.98)**	**1.34 (1.22, 1.48)**
Self-pay	**0.60 (0.55, 0.65)**	**0.66 (0.62, 0.70)**	**0.47 (0.40, 0.56)**	**1.44 (1.16, 1.80)**
Missing	**1.87 (1.74, 2.01)**	**1.69 (1.60, 1.78)**	**1.32 (1.20, 1.45)**	**1.23 (1.10, 1.37)**
**Elixhauser AHRQ weighted comorbidity score**^**e**^	**2.34 (2.28, 2.41)**	**1.78 (1.75, 1.80)**	**1.60 (1.56, 1.63)**	**1.17 (1.15, 1.20)**
**AUC**	0.86	**–**	0.86	**–**
**R**^**2**^	**–**	0.33	**–**	**–**

^a^Adjusted odds ratio from mixed-effect logistic regression model (clustering on one-digit zip-code).

^b^Adjusted exponentiated coefficients (mixed-effect exponential regression model clustering on one-digit zip-code) relating to change in the ratio of expected maximum length of hospital stay (i.e., “male” coefficient is the ratio of the expected max LOS for males over expected max LOS for females, so max LOS is 16% greater for males than for females).

^c^Adjusted hazard ratios from Cox-Proportional Hazard regression model.

^d^Adjusted change in outcome for every 10 year increase in age.

^e^Adjusted change in outcome for every 10 point increase in ECI.

^f^*p* values on the boundary of significance: Hospitalization gender other: 0.0503, max LOS gender other: 0.08.

Bold indicates statistical significance at the 5% level (i.e., *p* value < 0.05). Italic indicates *p* values are on the boundary of statistical significance (i.e., 0.05)

Compared with NH Whites, NH Blacks/African Americans had significantly longer hospital LOS (${e}^{\widehat{\beta }}$ 1.13; 95% CI [1.08, 1.19]), and were significantly more likely to be ventilator dependent (aOR 1.31; 95% CI [1.21, 1.43]) or die (aHR 1.22; 95% CI [1.13, 1.32]). Other race groups showed significantly higher associations with ventilator dependence and death compared with NH Whites (ventilator dependence aOR 1.72; death aHR 1.58). Hispanics/Latinos had lower odds of hospitalization (aOR 0.81; 95% CI [0.77, 0.86]), lower LOS (maximum LOS ${e}^{\widehat{\beta }}$: 0.88; 95% CI [0.85, 0.92]), and a lower hazard of death (aHR 0.89; 95% CI [0.82, 0.97]) compared with NH Whites. There was no evidence that Hispanics/Latinos had significantly higher odds of ventilator dependence (aOR: 1.09; 95% CI [1.00, 1.19]). All logistic models were classified with an AUC of 0.86. The exponential model explained 33% of the variation in maximum LOS.

### Racial disparities with comparable ECI scores

**Figure 2:**
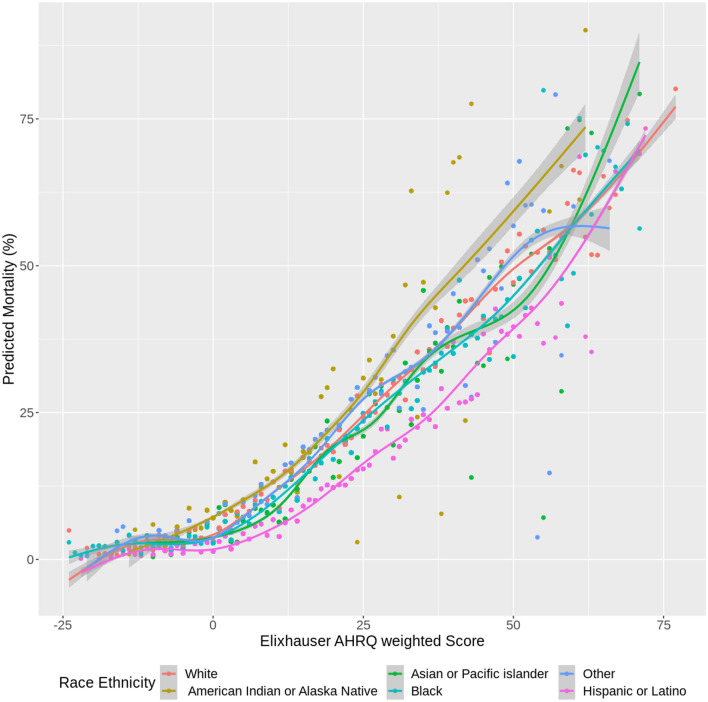
Predicted mortality versus Elixhauser AHRQ weighted score, among COVID-19 infected patients (by race).

**Figure 3:**
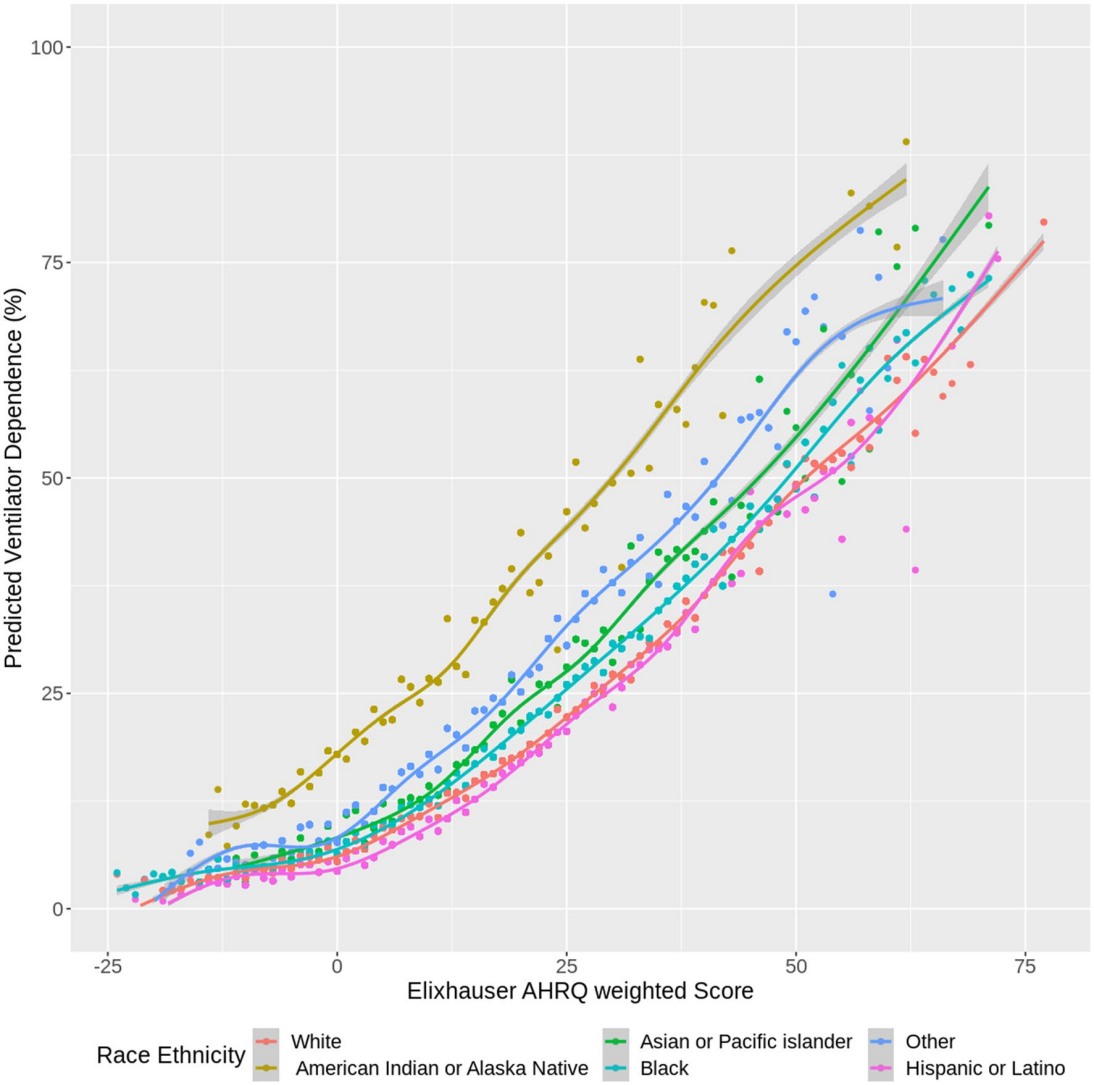
Predicted ventilator dependence versus Elixhauser AHRQ weighted score, among COVID-19 infected patients (by race).

Stratified analyses (in Supplemental Tables 4, 5, 6, and 7, Figs. 2 and 3, and Supplemental Figs. 1 and 2) showed differences among the outcomes. Although weighted ECI scores were comparable among races, we observed significant disparities in outcomes of COVID-19 complications. Compared with NH Whites, NH Blacks had longer hospital LOS (${e}^{\widehat{\beta }}$: 1.20; 95% CI [1.01, 1.43] for ECI = 1–4; 1.11; 95% CI [1.04, 1.17 for ECI of 5 or higher); were more likely to be ventilator dependent (aOR: 1.85; 95% CI [1.30, 2.64] for ECI = 0; 1.23; 95% CI [1.12, 1.35] for ECI of 5 or higher); and were more likely to die (aOR: 1.47; 95% CI [0.95, 2.27] for ECI = 0; 1.13; 95% CI [1.02, 1.25] for ECI of 5 or higher). Compared with NH Whites, AI/ANs had higher odds of hospitalization for ECI = 0 (aOR: 2.30; 95% CI [1.75, 3.02]) but lower odds of hospitalization for ECI of 5 or higher (aOR: 0.76; 95% CI [0.57, 1.02]); longer hospital LOS for ECI = 0 (${e}^{\widehat{\beta }}$: 2.75; 95% CI [2.28, 3.32]); a higher risk of death (aOR: 3.34; 95% CI [1.17, 9.56]) for ECI of less than 0; aOR: 5.77; 95% CI [3.07, 10.83] for ECI = 0; aOR: 2.69; 95% CI [0.87, 8.31] for ECI = 1–4); and higher odds of ventilator dependence across all ECI categories. Hispanics had a lower risk of death across all ECI categories except for ECI = 0, lower odds of hospitalization across all ECI categories, shorter hospital LOS for ECI of 5 or higher, and higher odds of ventilator dependence for ECI = 0 but lower odds of ventilator dependence for ECI = 1–4. Compared with NH Whites, patients of NH other or unknown race had longer LOS for all ECI categories except for ECI = 0 (aOR: 0.91; 95% CI [0.83, 0.99]), higher odds of invasive ventilator dependence across all ECI categories, and higher odds of death for ECI = 0 (aOR: 1.81; 95% CI [1.12, 2.91]) and ECI of 5 or higher (aOR: 1.27; 95% CI [1.11, 1.44]).

## Discussion

This study answers the question of whether racial disparities in COVID-19 outcomes exist despite comparable ECIs among NH Black, Hispanic, AI/AN, and White patients. To our knowledge, it is one of the largest systematic evaluations in the US of racial and ethnic differences in survival outcomes stratified by ECI score for patients with COVID-19. Our analyses revealed significant racial disparities in health outcomes among COVID-19 patients with comparable ECI scores. In particular, compared with NH Whites, most race groups had higher risk for all outcomes (hospitalization, LOS, ventilation, and death), with greater clinical and statistical significance for AI/ANs and NH Blacks. For example, using adjusted estimates, NH Blacks had longer LOS and higher odds of both ventilator dependence and death compared with NH Whites. NH Blacks and Native Americans were at increased risk for complications and death from COVID-19 compared with NH Whites.

Previous studies suggest that racial disparities in COVID-19 incidence and mortality can be explained by the complex interaction of inequities in social determinants of health, including access to health care^2,39,40^, poverty^40,41^, systemic racism^2,40^, socioeconomic status^2^, lack of testing for SARS-CoV-2 infection^39,42^, discrimination^2^, and virus exposure due to employment in essential-worker occupations^43,44^, all of which may be best viewed through a biopsychosocial framework akin to the weathering hypothesis, which posits that cumulative exposure to chronic stress can lead to accelerated aging by inducing physiologic changes that diminish the body’s ability to respond appropriately to acute stressors^45^. Preliminary investigations suggest that a higher prevalence of medical comorbidities explains the clinical differences in outcomes among patients with COVID-19^7,17,46–48^. Yet in our analysis of the 4 above-mentioned outcomes stratified by ECI AHRQ-weighted group, we still observed significant racial disparities in COVID-19 complications. Contrary to previous studies^7,17,46,49^, our analysis showed that for all races, the probability of hospitalization due to COVID-19 increased in unison with an increasing ECI. Accordingly, our findings contest arguments that NH Black and AI/AN patients are dying from COVID-19 at higher rates than their NH White counterparts because they have more comorbidities.

After adjustment for predictive association with our chief outcomes, our analysis revealed a higher risk for all 4 outcomes (hospitalization, LOS, ventilation, and death) among older patients, men (compared with women), patients with higher ECI scores, and patients covered by Medicare or Medicaid (compared with those covered by private insurance). These findings align with patterns identified in previous studies of cohorts ranging in size from 191 to 11,210^7,46^.

Disaggregation by race and ethnicity of the analysis of all 4 primary outcomes uncovered 3 overarching disparities while controlling for comorbidity. First, we found that APIs, NH Blacks, and patients of NH other or unknown race had a higher risk for all outcomes. This aligns with previous findings on racial disparities for NH Blacks for hospitalization^50^, mortality^19^, and ventilation^7^, and raises questions about the intersection of anti-Asian discrimination and xenophobia with health outcomes for API patients^51^. Secondly, our findings showed that, compared with NH Whites, AI/AN patients had a higher risk of death and higher odds of ventilator dependence but lower odds of hospitalization and a trend toward lower LOS for ECI of 5 or higher. These disproportionalities may be understood by the transfer of patients from Indian Health Service (IHS) facilities to non-IHS facilities, as IHS facilities are commonly ill-equipped to care for AI/AN patients with COVID-19 (e.g., they may lack invasive ventilation equipment)^52^. Third, our analysis showed that, compared with NH Whites, Hispanics/Latinos had a lower risk for death, hospitalization, and LOS, but higher odds of ventilator dependence for ECI = 0. Although these findings contradict epidemiological studies that have found a higher risk of COVID-19–related deaths within Hispanic/Latino communities^53,54^, they align with the “Hispanic epidemiological paradox,” which suggests that, although the socioeconomic characteristics of Hispanics/Latinos are similar to those of NH Blacks, comorbidity, mortality, and longevity outcomes in this subpopulation mirror or exceed those of NH Whites^55^.

Our data clearly show that a higher percentage of older patients were NH White and a higher percentage of younger patients were Hispanic/Latino (Supplemental Fig. 3). Other studies have found that, compared with NH Whites, Hispanic/Latino patients with COVID-19 tend to be younger^56^ and that older Hispanic/Latino patients with COVID-19 may have a higher risk for death^57,58^. Recent reports of higher COVID-19 death rates among older Hispanic/Latino populations^57^ and higher COVID-19 hospitalization rates among Hispanic/Latino children^59^ may challenge the “Hispanic paradox.” To better address the needs of the Hispanic/Latino population, future researchers should employ additional data disaggregation to address this question.

Lastly, our results indicate that older patients and individuals with higher ECI scores had an increased risk of death from COVID-19. Likewise, men compared with women, all races (except Hispanics/Latinos) compared with NH Whites, and patients with all other health insurance types compared with those with private insurance had an increased likelihood of death. These results are supported by recent findings of higher COVID-19 fatality rates among men, older persons, and patients with a disproportionate burden of comorbidities^60,61^. Emerging literature also points to an association of minority status and insurance type with poor COVID-19 outcomes^7^. Our logistic regression findings reveal similar associations with minority status and insurance type for hospitalizations, death, ventilator dependence, and hospital LOS.

This study has potential limitations. Some of the outcomes and predictors were identified by medical record codes (i.e., ICD and LOINC) that are known to limit the specificity of a study. However, we additionally applied a variety of alternative methods, such as text matching, to provide an additional net with which to capture all possible indications in the data. Medical histories were only available going back 5 years on qualifying patients included in the cohort. Our study included only patients who sought treatment for COVID-19. It is important to note that medically underserved and minority populations without insurance may not seek testing and treatment for COVID-19^62^, which has implications for both Hispanics/Latinos and NH Blacks, who are 2–3 times more likely to be uninsured compared with their NH White counterparts^63^. In addition, because (1) the data we analyzed included only individuals who had accessed health care services, and (2) post-mortem COVID testing is not routinely done, we may have underestimated the death rate among Hispanics/Latinos. Lastly, social variables that could play a potential confounding role in our study were not captured in the EHR data that we analyzed and thus were not included in the multilevel analyses.

## Conclusion

Compared with NH White patients with similar ECI scores, NH Black patients had significantly higher LOS and odds of ventilator dependence and death, while AI/AN patients were more likely to have worse indications across all 4 outcomes analyzed: hospitalization, LOS, ventilation, and death. COVID-19 has laid bare an imperative to investigate its negative health outcomes that may be exacerbated by a complex interplay of social, environmental, and behavioral factors faced by indigenous, Hispanic/Latino, and NH Black communities^31^, indicating a need for upstream intervention at patient, community, and policy levels to close the health equity gap.

## Supplementary Information

Supplementary Information

## Data Availability

The datasets generated and/or analyzed during the current study are not publicly available due to restrictions by Cerner, the owner of the data. Data may be accessed by signing a data-sharing agreement with Cerner and covering any costs that may be involved.
