# Supplementary material for: Racial disparities in COVID-19 outcomes exist despite comparable Elixhauser comorbidity indices between Blacks, Hispanics, Native Americans, and Whites
Source: Sci Rep. 2021 Apr 22;11:8738. doi: 10.1038/s41598-021-88308-2 (PMC8062526; doi:10.1038/s41598-021-88308-2)
Supplement: Supplementary file 1 — Supplementary Information [file 41598_2021_88308_MOESM1_ESM.docx]

**Supplemental Table 1:** List of codes identifying invasive ventilator dependence

| Code Type | Code |
| --- | --- |
| CPT-4 (HCPCS Level I) | 31500, 31600, 31601, 31603, 31605, 31610, 31615, 31820, 31825, 31830 |
| ICD-9-CM Diagnoses | 519, 519.02, 519.09, V44.0, V46.11, V55.0 |
| ICD-9-CM Procedures | 31.1, 31.74, 96.04, 97.23 |
| ICD-10-CM | J95.0, J95.00, J95.01, J95.02, J95.03, J95.04, J95.09, Z43.0, Z93.0, Z99.11 |
| ICD-10-PCS | 0B110F4,  0B113F4, 0B21XEZ, 0B21XFZ, 0BH13EZ, 0BH17EZ, 0BH18EZ, 0BP10FZ, 0BP1XFZ, 0BW10FZ, 0BW13FZ, 0BW18FZ, 0BW1XFZ |
| LOINC | 19834-1, 20058-4, 20077-4, 20079-0, 20112-9, 20116-0, 33438-3, LA11274-0 |
| SNOMED CT | 2267008, 6774004, 21619009, 55622001, 59006007, 68033004, 82872004, 112798008,  129121000, 161685006, 173070006, 232613003, 232685002, 274969000, 302108003,  302323001, 307007002, 348576000, 385858000, 448621002, 709146000 |

**Supplemental Table 2:** List of chronic diseases involved in Elixhauser comorbidity score, with corresponding ICD-10 codes

| Chronic Disease | ICD-10 Codes |
| --- | --- |
| Congestive heart failure | I09.9, I11.0, I13.0, I13.2, I25.5, I42.0, I42.5 - I42.9, I43.x, I50.x, P29.0 |
| Cardiac arrhythmias | I44.1 - I44.3, I45.6, I45.9, I47.x - I49.x, R00.0, R00.1, R00.8, T82.1, Z45.0, Z95.0 |
| Valvular disease | A52.0, I05.x - I08.x, I09.1, I09.8, I34.x - I39.x, Q23.0 - Q23.3, Z95.2 - Z95.4 |
| Pulmonary circulation disorders | I26.x, I27.x, I28.0, I28.8, I28.9 |
| Peripheral vascular disorders | I70.x, I71.x, I73.1, I73.8, I73.9, I77.1, I79.0, I79.2, K55.1, K55.8, K55.9, Z95.8, Z95.9 |
| Hypertension: uncomplicated | I10.x |
| Hypertension: complicated | I11.x - I13.x, I15.x |
| Paralysis | G04.1, G11.4, G80.1, G80.2, G81.x, G82.x, G83.0 - G83.4, G83.9 |
| Other neurological disorders | G10.x - G13.x, G20.x - G22.x, G25.4, G25.5, G31.2, G31.8, G31.9, G32.x, G35.x - G37.x, G40.x, G41.x, G93.1, G93.4, R47.0, R56.x |
| Chronic pulmonary disease | I27.8, I27.9, J40.x - J47.x, J60.x - J67.x, J68.4, J70.1, J70.3 |
| Diabetes, uncomplicated | E10.0, E10.1, E10.9, E11.0, E11.1, E11.9, E12.0, E12.1, E12.9, E13.0, E13.1, E13.9, E14.0, E14.1, E14.9 |
| Diabetes, complicated | E10.2 - E10.8, E11.2 - E11.8, E12.2 - E12.8, E13.2 - E13.8, E14.2 - E14.8 |
| Hypothyroidism | E00.x - E03.x, E89.0 |
| Renal failure | I12.0, I13.1, N18.x, N19.x, N25.0, Z49.0 - Z49.2, Z94.0, Z99.2 |
| Liver disease | B18.x, I85.x, I86.4, I98.2, K70.x, K71.1, K71.3 - K71.5, K71.7, K72.x - K74.x, K76.0, K76.2 - K76.9, Z94.4 |
| Peptic ulcer disease, excluding bleeding | K25.7, K25.9, K26.7, K26.9, K27.7, K27.9, K28.7, K28.9 |
| AIDS/HIV | B20.x - B22.x, B24.x |
| Lymphoma | C81.x - C85.x, C88.x, C96.x, C90.0, C90.2 |
| Metastatic cancer | C77.x-C80.x |
| Solid tumor without metastasis | C00.x - C26.x, C30.x - C34.x, C37.x - C41.x, C43.x, C45.x - C58.x, C60.x - C76.x, C97.x |
| Rheumatoid arthritis/collagen vascular diseases | L94.0, L94.1, L94.3, M05.x, M06.x, M08.x, M12.0, M12.3, M30.x, M31.0 - M31.3, M32.x - M35.x, M45.x, M46.1, M46.8, M46.9 |
| Coagulopathy | D65 - D68.x, D69.1, D69.3 - D69.6 |
| Obesity | E66.x |
| Weight loss | E40.x - E46.x, R63.4, R64 |
| Fluid and electrolyte disorders | E22.2, E86.x, E87.x |
| Blood loss anemia | D50.0 |
| Deficiency anemia | D50.8, D50.9, D51.x - D53.x |
| Alcohol abuse | F10, E52, G62.1, I42.6, K29.2, K70.0, K70.3, K70.9, T51.x, Z50.2, Z71.4, Z72.1 |
| Drug abuse | F11.x - F16.x, F18.x, F19.x, Z71.5, Z72.2 |
| Psychoses | F20.x, F22.x - F25.x, F28.x, F29.x, F30.2, F31.2, F31.5 |
| Depression | F20.4, F31.3 - F31.5, F32.x, F33.x, F34.1, F41.2, F43.2 |

**Supplemental Table 3:** Adjusted hospitalization, maximum length of hospital stay, dependence on invasive ventilator, and death (logistic model) from COVID-19

| Variables | Hospitalization | Maximum Length of Stay | Invasive Ventilator Dependence | Deceased |
| --- | --- | --- | --- | --- |
|  | aOR (95% CI) | $e^{\hat{\beta}}$ (95% CI) | aOR (95% CI) | aOR (95% CI) |
| Age (years) | **1.30 (1.28, 1.32)** | **1.30 (1.29, 1.31)** | **1.16 (1.14, 1.18)** | **1.69 (1.65, 1.74)** |
| Gender |  |  |  |  |
| Female | 1 [Reference] | 1 [Reference] | 1 [Reference] | 1 [Reference] |
| Male | **1.23 (1.18, 1.28)** | **1.22 (1.18, 1.26)** | **1.55 (1.46, 1.64)** | **1.54 (1.44, 1.64)** |
| Other | 1.60 (1.00, 2.57) | 1.37 (0.96, 1.95) | 1.50 (0.82, 2.75) | 1.60 (0.77, 3.31) |
| Race and Ethnicity |  |  |  |  |
| Non-Hispanic White | 1 [Reference] | 1 [Reference] | 1 [Reference] | 1 [Reference] |
| Non-Hispanic American Indian or Alaska Native | **1.21 (1.03, 1.43)** | **1.32 (1.16, 1.51)** | **3.49 (2.87, 4.25)** | **2.26 (1.74, 2.92)** |
| Non-Hispanic Asian or Pacific Islander | 1.08 (0.95, 1.23) | **1.15 (1.05, 1.27)** | **1.44 (1.22, 1.69)** | 1.06 (0.87, 1.29) |
| Non-Hispanic Black or African American | 1.02 (0.95, 1.08) | **1.13 (1.08, 1.19)** | **1.31 (1.21, 1.43)** | **1.19 (1.08, 1.30)** |
| Non-Hispanic Other | 0.99 (0.91, 1.06) | 1.06 (1.00, 1.12) | **1.72 (1.56, 1.90)** | **1.41 (1.25, 1.58)** |
| Hispanic or Latino | **0.81 (0.77, 0.86)** | **0.88 (0.85, 0.92)** | 1.09 (1.00, 1.19) | **0.84 (0.76, 0.93)** |
| Insurance^3^ |  |  |  |  |
| Private | 1 [Reference] | 1 [Reference] | 1 [Reference] | 1 [Reference] |
| Government/Misc | 1.09 (0.98, 1.22) | **1.11 (1.02, 1.21)** | 0.93 (0.79, 1.09) | **1.27 (1.04, 1.54)** |
| Medicaid | **1.64 (1.54, 1.74)** | **1.65 (1.58, 1.74)** | **1.11 (1.01, 1.22)** | **1.43 (1.24, 1.64)** |
| Medicare | **1.51 (1.41, 1.62)** | **1.50 (1.42, 1.58)** | **0.90 (0.82, 0.98)** | **1.39 (1.25, 1.55)** |
| Self-Pay | **0.60 (0.55, 0.65)** | **0.66 (0.62, 0.70)** | **0.47 (0.40, 0.56)** | 0.91 (0.73, 1.14) |
| Missing | **1.87 (1.74, 2.01)** | **1.69 (1.60, 1.78)** | **1.32 (1.20, 1.45)** | **1.50 (1.33, 1.71)** |
| Elixhauser AHRQ weighted Comorbidity Score | **2.34 (2.28, 2.41)** | **1.78 (1.75, 1.80)** | **1.60 (1.56, 1.63)** | **1.52 (1.49, 1.56)** |
| AUC | 0.86 | **-** | 0.86 | 0.86 |
| R^2^ | **-** | 0.33 | **-** | - |

**Supplemental Table 4:** Adjusted variables impact on likelihood of death among COVID-19 infected patients, stratified by Elixhauser AHRQ weighted Comorbidity Index

| Variables | <0 | 0 | 1-4 | >=5 |
| --- | --- | --- | --- | --- |
|  | aOR (95% CI) | aOR (95% CI) | aOR (95% CI) | aOR (95% CI) |
| Age (years) | **1.91 (1.68, 2.17)** | **2.29 (2.05, 2.55)** | **1.87 (1.65, 2.13)** | **1.60 (1.55, 1.65)** |
| Gender |  |  |  |  |
| Female | 1 [Reference] | 1 [Reference] | 1 [Reference] | 1 [Reference] |
| Male | **1.36 (1.01, 1.83)** | **2.24 (1.68, 2.97)** | 1.35 (0.99, 1.85)^1^ | **1.54 (1.43, 1.66)** |
| Race and Ethnicity |  |  |  |  |
| Non-Hispanic White | 1 [Reference] | 1 [Reference] | 1 [Reference] | 1 [Reference] |
| Non-Hispanic American Indian or Alaska Native | **3.34 (1.17, 9.56)** | **5.77 (3.07, 10.83)** | *2.69 (0.87, 8.31)^1^* | 1.09 (0.76, 1.57) |
| Non-Hispanic Asian or Pacific Islander | 0.47 (0.14, 1.53) | 0.89 (0.37, 2.18) | 0.61 (0.18, 2.08) | 1.10 (0.90, 1.35) |
| Non-Hispanic Black or African American | 1.05 (0.70, 1.55) | *1.47 (0.95, 2.27)^1^* | 0.89 (0.58, 1.37) | **1.13 (1.02, 1.25)** |
| Non-Hispanic Other | 0.98 (0.58, 1.67) | **1.81 (1.12, 2.91)** | 1.51 (0.92, 2.48) | **1.27 (1.11, 1.44)** |
| Hispanic or Latino | **0.56 (0.35, 0.88)** | 0.80 (0.52, 1.24) | **0.55 (0.34, 0.87)** | **0.87 (0.78, 0.97)** |
| Insurance |  |  |  |  |
| Private | 1 [Reference] | 1 [Reference] | 1 [Reference] | 1 [Reference] |
| Government/Misc | 1.29 (0.53, 3.16) | **2.76 (1.49, 5.11)** | -^1^ | **1.26 (1.01, 1.56)** |
| Medicaid | 1.39 (0.80, 2.42) | **1.90 (1.10, 3.27)** | **2.14 (1.17, 3.91)** | **1.33 (1.14, 1.56)** |
| Medicare | **1.65 (1.05, 2.59)** | **2.67 (1.73, 4.14)** | **2.12 (1.29, 3.50)** | **1.31 (1.17, 1.48)** |
| Self-Pay | 1.05 (0.46, 2.39) | 0.90 (0.46, 1.74) | 2.09 (0.96, 4.58)^1^ | 1.11 (0.85, 1.46) |
| Missing | 1.70 (0.99, 2.92)^1^ | **2.14 (1.33, 3.45)** | **1.85 (1.02, 3.38)** | **1.39 (1.21, 1.59)** |

^1^ p-values on the boundary of significance: <0 missing: 0.055, 0 NH Black: 0.08, 1-4 male: 0.058, 1-4 NH AIAN: 0.09, 1-4 self-pay: 0.06

**Supplemental Table 5:** Adjusted variables impact on likelihood of hospitalization among COVID-19 infected patients, stratified by Elixhauser AHRQ weighted Comorbidity Index

| Variables | <0 | 0 | 1-4 | >=5 |
| --- | --- | --- | --- | --- |
|  | aOR (95% CI) | aOR (95% CI) | aOR (95% CI) | aOR (95% CI) |
| Age (years) | **1.15 (1.12, 1.19)** | **1.26 (1.23, 1.30)** | **1.36 (1.31, 1.42)** | **1.24 (1.21, 1.26)** |
| Gender |  |  |  |  |
| Female | 1 [Reference] | 1 [Reference] | 1 [Reference] | 1 [Reference] |
| Male | **1.28 (1.17, 1.40)** | **1.16 (1.07, 1.26)** | **1.4 (1.22, 1.60)** | **1.37 (1.28, 1.47)** |
| Race and Ethnicity |  |  |  |  |
| Non-Hispanic White | 1 [Reference] | 1 [Reference] | 1 [Reference] | 1 [Reference] |
| Non-Hispanic American Indian or Alaska Native | 0.91 (0.62, 1.33) | **2.30 (1.75, 3.02)** | 1.08 (0.61, 1.91) | *0.76 (0.57, 1.02)^1^* |
| Non-Hispanic Asian or Pacific Islander | 1.23 (0.91, 1.65) | 0.97 (0.75, 1.27) | 1.28 (0.82, 2.01) | 1.12 (0.90, 1.40) |
| Non-Hispanic Black or African American | 0.97 (0.85, 1.11) | **0.78 (0.67, 0.90)** | 1.07 (0.88, 1.31) | 0.98 (0.88, 1.09) |
| Non-Hispanic Other | 1.13 (0.95, 1.34) | 0.92 (0.79, 1.07) | 1.07 (0.82, 1.38) | 1.10 (0.95, 1.26) |
| Hispanic or Latino | **0.82 (0.72, 0.93)** | **0.83 (0.73, 0.93)** | **0.78 (0.65, 0.94)** | **0.79 (0.72, 0.88)** |
| Insurance |  |  |  |  |
| Private | 1 [Reference] | 1 [Reference] | 1 [Reference] | 1 [Reference] |
| Government/Misc | 1.18 (0.92, 1.51) | **1.28 (1.04, 1.57)** | 1.10 (0.75, 1.60) | 1.11 (0.90, 1.37) |
| Medicaid | **1.48 (1.30, 1.68)** | **1.92 (1.70, 2.18)** | **1.77 (1.46, 2.16)** | **1.34 (1.19, 1.50)** |
| Medicare | **1.85 (1.59, 2.16)** | **2.49 (2.10, 2.95)** | **1.56 (1.26, 1.95)** | **1.28 (1.15, 1.42)** |
| Self-Pay | **0.70 (0.59, 0.84)** | **0.66 (0.57, 0.77)** | **0.70 (0.53, 0.92)** | **0.72 (0.61, 0.84)** |
| Missing | **1.76 (1.50, 2.06)** | **2.37 (2.06, 2.74)** | **2.13 (1.67, 2.70)** | **1.60 (1.42, 1.81)** |

^1^ p-values on the boundary of significance: >=5 NH AIAN: 0.07

**Supplemental Table 6:** Adjusted variables impact on likelihood of invasive ventilator dependence among COVID-19 infected patients, stratified by Elixhauser AHRQ weighted Comorbidity Index

| Variables | <0 | 0 | 1-4 | >=5 |
| --- | --- | --- | --- | --- |
|  | aOR (95% CI) | aOR (95% CI) | aOR (95% CI) | aOR (95% CI) |
| Age (years) | **1.21 (1.12, 1.29)** | **1.58 (1.48, 1.68)** | **1.24 (1.14, 1.33)** | **1.07 (1.05, 1.09)** |
| Gender |  |  |  |  |
| Female | 1 [Reference] | 1 [Reference] | 1 [Reference] | 1 [Reference] |
| Male | **1.58 (1.30, 1.92)** | **1.77 (1.46, 2.15)** | **1.49 (1.18, 1.89)** | **1.54 (1.44, 1.64)** |
| Race and Ethnicity |  |  |  |  |
| Non-Hispanic White | 1 [Reference] | 1 [Reference] | 1 [Reference] | 1 [Reference] |
| Non-Hispanic American Indian or Alaska Native | **4.51 (2.21, 9.23)** | **12.28 (7.51, 20.09)** | **3.53 (1.59, 7.83)** | **1.46 (1.10, 1.95)** |
| Non-Hispanic Asian or Pacific Islander | **1.82 (1.05, 3.14)** | *1.67 (0.91, 3.05)^1^* | 0.99 (0.44, 2.24) | **1.38 (1.15, 1.65)** |
| Non-Hispanic Black or African American | 1.24 (0.93, 1.65) | **1.85 (1.30, 2.64)** | 0.87 (0.61, 1.23) | **1.23 (1.12, 1.35)** |
| Non-Hispanic Other | **1.76 (1.25, 2.48)** | **2.04 (1.40, 2.97)** | **1.80 (1.23, 2.63)** | **1.59 (1.42, 1.78)** |
| Hispanic or Latino | 1.00 (0.73, 1.35) | **1.65 (1.20, 2.27)** | *0.73 (0.52, 1.03)^1^* | 1.06 (0.97, 1.17) |
| Insurance |  |  |  |  |
| Private | 1 [Reference] | 1 [Reference] | 1 [Reference] | 1 [Reference] |
| Government/Misc | 1.05 (0.63, 1.75) | **1.82 (1.19, 2.78)** | 1.02 (0.52, 1.99) | 0.86 (0.71, 1.05) |
| Medicaid | 1.16 (0.86, 1.56) | **1.88 (1.38, 2.55)** | **1.57 (1.08, 2.26)** | 0.97 (0.86, 1.09) |
| Medicare | 1.04 (0.76, 1.42) | **1.76 (1.27, 2.44)** | *1.39 (0.96, 2.00)^1^* | **0.90 (0.81, 0.99)** |
| Self-Pay | 0.61 (0.38, 1.00) | **0.49 (0.31, 0.76)** | 0.72 (0.39, 1.31) | **0.59 (0.48, 0.73)** |
| Missing | **1.67 (1.21, 2.32)** | **2.64 (1.95, 3.56)** | **1.62 (1.07, 2.46)** | **1.13 (1.01, 1.26)** |

^1^ p-values on the boundary of significance: 0 NH API: 0.095, 1-4 Hispanic/Latino: 0.08, 1-4 Medicare: 0.08

**Supplemental Table 7:** Adjusted variables impact on maximum length of stay among COVID-19 infected patients, stratified by Elixhauser AHRQ weighted Comorbidity Index

| Variables | <0 | 0 | 1-4 | >=5 |
| --- | --- | --- | --- | --- |
|  | $e^{\hat{\beta}}$ (95% CI) | $e^{\hat{\beta}}$ (95% CI) | $e^{\hat{\beta}}$ (95% CI) | $e^{\hat{\beta}}$ (95% CI) |
| Age (years) | **1.19 (1.16, 1.22)** | **1.22 (1.20, 1.24)** | **1.36 (1.31, 1.41)** | **1.19 (1.18, 1.21)** |
| Gender |  |  |  |  |
| Female | 1 [Reference] | 1 [Reference] | 1 [Reference] | 1 [Reference] |
| Male | **1.30 (1.20, 1.41)** | **1.09 (1.04, 1.14)** | **1.27 (1.13, 1.42)** | **1.29 (1.24, 1.35)** |
| Race and Ethnicity |  |  |  |  |
| Non-Hispanic White | 1 [Reference] | 1 [Reference] | 1 [Reference] | 1 [Reference] |
| Non-Hispanic American Indian or Alaska Native | 1.06 (0.75, 1.50) | **2.75 (2.28, 3.32)** | 1.40 (0.85, 2.32) | 0.88 (0.73, 1.08) |
| Non-Hispanic Asian or Pacific Islander | 1.15 (0.88, 1.51) | 1.04 (0.88, 1.23) | 1.18 (0.80, 1.73) | **1.20 (1.06, 1.36)** |
| Non-Hispanic Black or African American | 1.08 (0.95, 1.22) | 0.95 (0.87, 1.04) | **1.20 (1.01, 1.43)** | **1.11 (1.04, 1.17)** |
| Non-Hispanic Other | **1.18 (1.01, 1.38)** | **0.91 (0.83, 0.99)** | *1.23 (0.99, 1.54)^1^* | **1.19 (1.11, 1.29)** |
| Hispanic or Latino | 0.92 (0.82, 1.03) | *0.93 (0.87, 1.00)^1^* | 0.88 (0.75, 1.03) | **0.91 (0.86, 0.97)** |
| Insurance |  |  |  |  |
| Private | 1 [Reference] | 1 [Reference] | 1 [Reference] | 1 [Reference] |
| Government/Misc | *1.24 (0.99, 1.56)^1^* | *1.14 (1.00, 1.30)^1^* | 1.32 (0.95, 1.83) | 1.04 (0.92, 1.18) |
| Medicaid | **1.53 (1.36, 1.72)** | **1.69 (1.57, 1.82)** | **1.72 (1.45, 2.03)** | **1.26 (1.17, 1.36)** |
| Medicare | **2.03 (1.76, 2.33)** | **2.77 (2.45, 3.13)** | **1.61 (1.34, 1.95)** | **1.17 (1.10, 1.25)** |
| Self-Pay | **0.80 (0.69, 0.92)** | **0.87 (0.81, 0.94)** | **0.77 (0.62, 0.95)** | **0.74 (0.66, 0.83)** |
| Missing | **1.70 (1.47, 1.96)** | **1.78 (1.62, 1.96)** | **1.92 (1.56, 2.36)** | **1.36 (1.27, 1.46)** |

^1^ p-values on the boundary of significance: <0 Insure Govt/Misc: 0.057, 0 Hispanic/Latino: 0.059, 0 Insure Govt/Misc: 0.053, 1-4 Race other: 0.06

**Supplemental Figure 1:** Predicted hospitalization vs. Elixhauser AHRQ weighted score, among COVID-19 infected patients (by race)


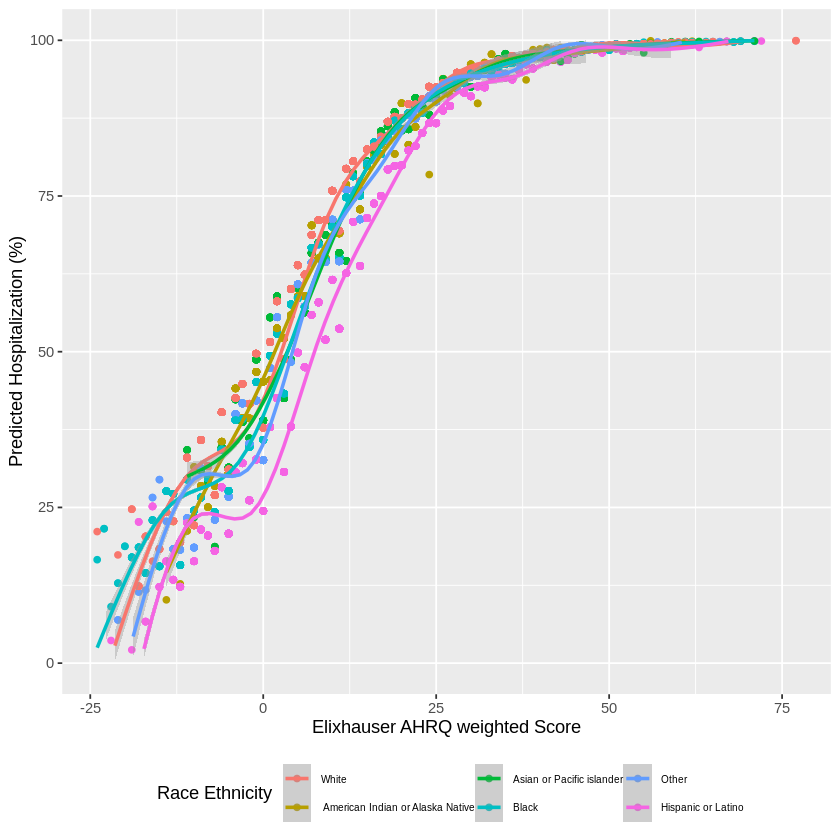


**Supplemental Figure 2:** Predicted maximum length of stay vs. Elixhauser AHRQ weighted score, among COVID-19 infected patients (by race)


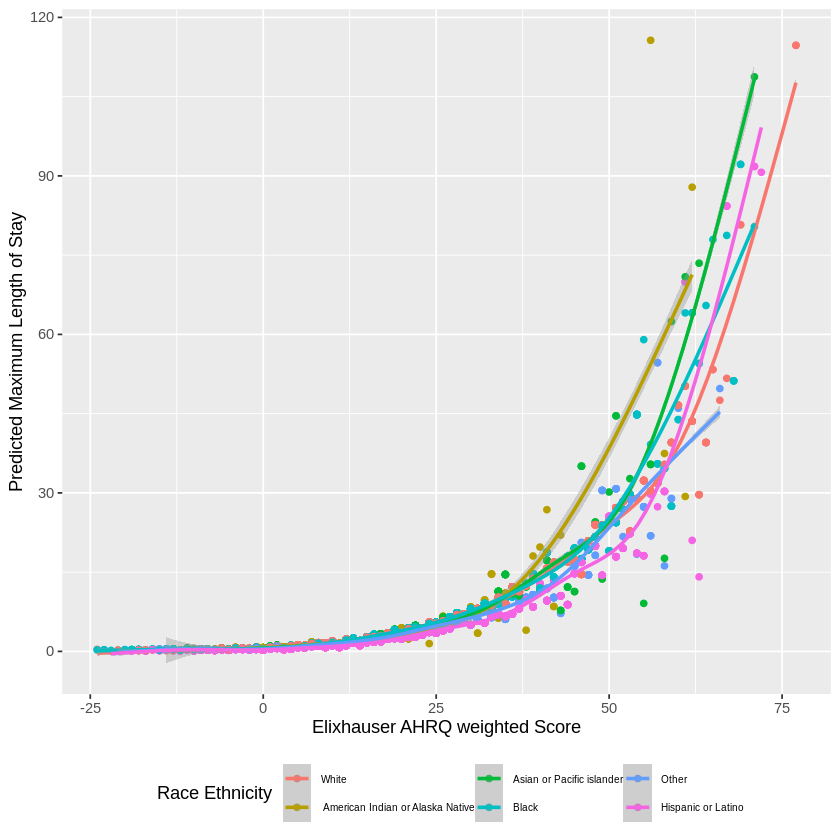


**Supplemental Figure 3:** Race and ethnicity distributions by age

**
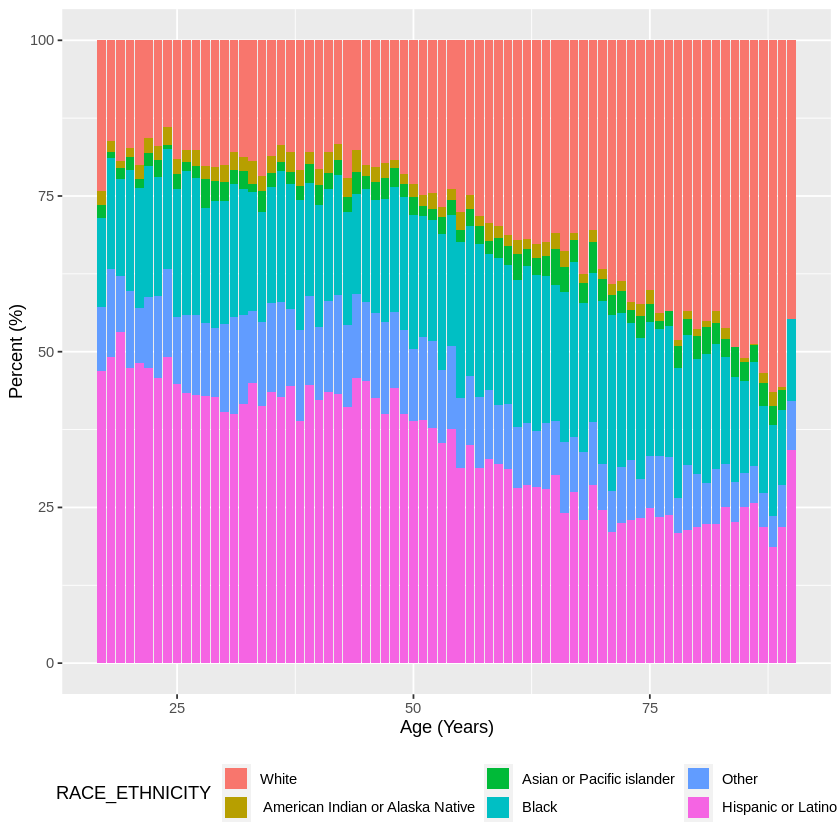
**
